# Reduced Expression of Inflammatory Genes in Deceased Donor Kidneys Undergoing Pulsatile Pump Preservation

**DOI:** 10.1371/journal.pone.0035526

**Published:** 2012-04-24

**Authors:** Valeria R. Mas, Kellie J. Archer, Catherine I. Dumur, Mariano J. Scian, Jihee L. Suh, Anne L. King, Megan E. Wardius, Julie A. Straub, Marc P. Posner, Kenneth Brayman, Daniel G. Maluf

**Affiliations:** 1 Translational Genomics Transplant Laboratory, University of Virginia, Charlottesville, Virginia, United States of America; 2 Department of Biostatistics, Virginia Commonwealth University, Richmond, Virginia, United States of America; 3 Department of Pathology, Virginia Commonwealth University, Richmond, Virginia, United States of America; 4 Transplant Division, Department of Surgery, Virginia Commonwealth University, Richmond, Virginia, United States of America; 5 Transplant Division, Department of Surgery, University of Virginia, Charlottesville, Virginia, United States of America; The University of Hong Kong, Hong Kong

## Abstract

**Background:**

The use of expanded criteria donor kidneys (ECD) had been associated with worse outcomes. Whole gene expression of pre-implantation allograft biopsies from deceased donor kidneys (DDKs) was evaluated to compare the effect of pulsatile pump preservation (PPP) vs. cold storage preservation (CSP) on standard and ECD kidneys.

**Methodology/Principal Findings:**

99 pre-implantation DDK biopsies were studied using gene expression with GeneChips. Kidneys transplant recipients were followed post transplantation for 35.8 months (range = 24–62). The PPP group included 60 biopsies (cold ischemia time (CIT)  = 1,367+/−509 minutes) and the CSP group included 39 biopsies (CIT = 1,022+/−485 minutes) (P<0.001). Donor age (42.0±14.6 vs. 34.1±14.2 years, P = 0.009) and the percentage of ECD kidneys (PPP = 35% vs. CSP = 12.8%, P = 0.012) were significantly different between groups. A two-sample t-test was performed, and probe sets having a P<0.001 were considered significant. Probe set level linear models were fit using cold ischemia time and CSP/PPP as independent variables to determine significant probe sets (P<0.001) between groups after adjusting for cold ischemia time. Thus, 43 significant genes were identified (P<0.001). Over-expression of genes associated with inflammation (CD86, CD209, CLEC4, EGFR2, TFF3, among others) was observed in the CSP group. Cell-to-cell signaling and interaction, and antigen presentation were the most important pathways with genes significantly over-expressed in CSP kidneys. When the analysis was restricted to ECD kidneys, genes involved in inflammation were also differentially up-regulated in ECD kidneys undergoing CSP. However, graft survival at the end of the study was similar between groups (P = 0.2). Moreover, the incidence of delayed graft function was not significant between groups.

**Conclusions/Significance:**

Inflammation was the most important up-regulated pattern associated with pre-implantation biopsies undergoing CSP even when the PPP group has a larger number of ECD kidneys. No significant difference was observed in delayed graft function incidence and graft function post-transplantation. These findings support the use of PPP in ECD donor kidneys.

## Introduction

Kidney transplantation (KT) represents the treatment of choice for patients with end-stage renal disease and provides survival benefit when compared with long term dialysis therapy, in terms of quality of life and life expectancy [1], [2]. With the goal of improving transplantation outcomes, donor and recipient selection criteria are evolving. Unfortunately, the national kidney waiting list continues to grow disproportionate to the number of donor organs available for transplantation. Median waiting times for kidney transplant in the US exceed 3 years in the absence of a living donor [3].

The increasing disparity between organ supply and demand challenges the transplantation community to maximize efforts and optimize the use of organs from all consented donors. Recent increases in graft availability from deceased donors have been a result of expansion of the donor acceptance criteria, including increasing use of older donors, donation-after-cardiac-death (DCD), and deceased donors with other characteristics that might be associated with increased risk of graft dysfunction [3]–[6]. To counteract the escalating discrepancy between organ availability and need, OPTN initiated policy in 2002 defining and providing guidelines for the use of expanded criteria donor (ECD) kidneys [7].

However, ECD kidneys had been often associated with worse short and long term renal function, and the limited nephron reserve might result in a relative risk of graft loss when compared with kidneys from standard criteria donors (SCD) [3]–[6]. Growing acceptance and use of ECD and DCD kidneys has been tempered by data suggesting that ECD kidneys have an increased susceptibility to ischemia-reperfusion injury, leading to higher rates of primary non-function; delayed graft function (DGF) and acute cellular rejection (ACR) [3]–[10]. These concerns have encouraged initiatives to qualitatively assess quality of these kidneys before transplantation by use of *ex-vivo* biopsies and machine preservation technology [11]–[13].

Furthermore, it is well-established that post-transplantation occurrences such as DGF and ACR are risk factors for poorer intermediate-term graft survival, increasing the burden of patients returning to dialysis therapy [2], [11], [14], [15]. According to UNOS data, the incidence of DGF is highest with DCD kidneys (44%), intermediate with ECD kidneys (33%), and lowest with SCD kidneys (21%) [2], [11], [14], [15].

From the time a patient is identified as a potential donor it is critical to maintain adequate organ perfusion and avoid hypoxemia. Currently, once a deceased donor kidney is recovered, there are two main methods of preservation including cold hypothermic storage preservation (CSP) and pulsatile perfusion preservation (PPP) [11]–[13], [16], [17]. Pulsatile perfusion preservation is being used in many organ procurement organizations with the goal of extending cold ischemia time (CIT) to optimize organ placement, and ultimately to improve transplant rates and organ quality. However, it is recognized that this practice has an increased transplant and acquisition cost.

More importantly, recent reports support the use of PPP demonstrating clinical benefit despite of the fact that the patho-physiological mechanisms involved in the “improved” graft function are still unclear. Assessment of the transcriptome in the donor organ itself is an appealing strategy to determine organ quality and predict subsequent graft performance, as molecular pathways may provide a comprehensive measurement of the individual graft's response to acute injury factors. In the current study, the transcriptome of 99 deceased donor pre-implantation biopsies of deceased donor kidney grafts preserved with CSP and PPP was evaluated.

## Materials and Methods

### Patients and samples

The study was conducted at Virginia Commonwealth University and at the University of Virginia after Institutional Review Board (IRB) approval was obtained at both institutions (VCU#HM11454, UVA 14849). Patients received a deceased donor kidney (DDK) transplant between January 2006 and January 2010. They were aware of the collection of biopsies and they signed a consent form (that includes description of risk associated with biopsies). They had the right to agree/deny the use of a pre-transplant biopsy. All organ donors (or their next of kin) consented for their samples to be used in research (as part of the overall protocol and consent for organ donation). Additionally, because the present research results are part of an overall study that includes the use of protocol biopsies and more than “minimal risks to the individual”, a Data and Safety Monitoring Board has been established for the study evaluation. Complications (expected and unexpected) are reported to the Institutional Review Boards (report of complications: between two weeks of occurrence, report of overall study performance: every three months) and to the study's sponsor as part of the yearly progress report. No living donors, HIV positive or re-transplantation patients were included in the study. Allograft biopsies from kidneys preserved using both cold preservation (CSP) and pump perfusion preservation (PPP) were included in the study. All the transplant recipients underwent the same immunosuppressant protocol that included a calcineurin inhibitor based plus mycophenolate mofetil and prednisone. Acute cellular rejection episodes were always treated with steroids boluses (three days followed by prednisone taper).

Kidney allograft tissue was obtained through an 18 gauge biopsy needle and all samples were placed in RNA*later* (Ambion) immediately after collection. Biopsies were collected at pre-implantation time (post-cold ischemia time; n = 99). Estimated GFR (eGFR) was calculated using the abbreviated Modification of Diet in Renal Disease (MDRD) formula [18]. Delayed graft function (DGF) was defined as the need of dialysis during the first 7 days post-kidney transplantation [14].

### RNA isolation, cDNA synthesis, and in vitro transcription for labeled cRNA probe

The sample preparation protocol follows the Affymetrix GeneChip® Expression Analysis Manual (Santa Clara, CA). Briefly, total RNA was reverse-transcribed using T7-polydT primer and converted into double-stranded cDNA (One-Cycle Target Labeling and Control Reagents, Affymetrix), with templates being used for an in vitro transcription reaction to yield biotin-labeled antisense cRNA. The labeled cRNA was chemically fragmented and made into the hybridization cocktail according to the Affymetrix GeneChip protocol, which was then hybridized to HG-U133A 2.0 GeneChips. The array image was generated by the high-resolution GeneChip® Scanner 3000 by Affymetrix®.

### Quality control and gene expression data analysis

GeneChip HG-U133Av2 arrays were hybridized for 99 pre-implantation samples. Quality control parameters that included scaling factor, average background, percent of probe sets called present, and the 3′∶5′ ratio for GAPDH and B-actin, were checked. The robust multiarray average method was used to obtain probe set expression summaries. Prior to performing statistical analyses, control probe sets and probe sets called absent across all 99 arrays were excluded, leaving 17,654 probe sets.

To identify probe sets differentially expressed between the pump (n = 60) and no pump (n = 39) groups, for each probe set a two-sample t-test was performed and probe sets having a *P*<0.001 were considered significant. Because there was a significant difference between the CSP and PPP groups with respect to cold ischemia time (CIT) (*P* = 0.0003), probe set level linear models were fit whereby expression was modeled using CIT and CSP/PPP as independent variables, to identify probe sets that were significantly different between the two groups after adjusting for CIT.

### Stratified analysis using cold ischemia time (CIT)

First, the dataset was restricted to patients receiving a graft with a CIT<1200 minutes (20 hours) and for each probe set a two-sample t-test was performed. Second, the dataset was restricted to graft with a CIT>1200 minutes and again, for each probe set a two-sample t-test was performed. For each analysis, probe sets having a *P*<0.001 were considered significant.

### S-Score algorithm for paired kidneys

Three pairs of Affymetrix HG-U133Av2 GeneChips were considered: 6K1 *vs.* 7K1b, 49K1 *vs.* 50K1, and 82-K *vs.* 83-K. For each pair of GeneChips, the probe level data were read into the R programming environment and the Affymetrix MAS5 detection call algorithm was applied. Probe sets that were absent in both GeneChips were removed from subsequent analysis. Thereafter, the S-Score algorithm [19], [20] was applied using the *S-Score* package in R [21]. The resulting *P*-values were used to estimate the false discovery rate using the q-value method [22]. Probe sets were considered significant when the *q*-value was less than 0.05. The log_2_ transformation was applied to probe set expression summaries obtained using the Affymetrix MAS5 algorithm for producing scatterplots and calculating fold changes.

### Interaction networks and functional analysis

Gene ontology and gene interaction analyses were executed using Ingenuity Pathways Analysis tools 9.0 (http://www.ingenuity.com).

### Validation of microarray results

We carried out a quantitative reverse transcriptase-“real time” PCR (QPCR) for CD86, CD209, and EGFR2 mRNAs in the same RNAs samples that were subjected to microarray study. Total RNA was subjected to reverse transcription using TaqMan® Reverse Transcription Reagents (Applied Biosystems, Foster City, CA) according to the manufacturer's protocol. QPCR reactions then were carried out using TaqMan® Gene Expression Assays (Applied Biosystems). Data was analyzed according to the comparative cycle threshold method and was normalized with a housekeeping gene (Glyceraldehyde 3-phosphate dehydrogenase (GAPDH)). Pearson's correlation coefficient (r) was calculated to examine the relation between microarray and QPCR results. *P*<0.05 were considered significant.

### Statistical analysis

Descriptive statistics were reported for demographic and clinical variables, including proportions for categorical variables and mean ± standard deviation for continuous variables.

## Results

### Donor and kidney biopsy characteristics

A total of 99 pre-implantation kidney graft biopsies from 92 deceased donors were studied. Seven donors provided paired kidneys. Patients were followed post transplantation for 35.8 months (range = 24–62). All biopsies were performed by the same surgical team, and consisted of a single 18 gauge needle biopsy taken from the cortex (2–15 mm deep) kidney upper pole, and right before implantation was started (at back-table and post-ischemia time). Samples were submerged immediately in RNA*later* (Ambion) and sent to the molecular laboratory. An additional sample from each kidney was sent for pathological evaluation.

No significant differences were observed in the histology of donor biopsies (pre-implantation) between groups (**Table S1**). Demographic and clinical information for the enrolled study patients were separated according to the preservation method used (PPP *vs.* CSP) as shown in **Table 1**. Sixty DDK were preserved with PPP (CIT = 1,367+/−509 minutes) and 39 DDK were preserved with CSP (CIT = 1,022+/−485 minutes) (*P*<0.001). Clinically, the two patient groups were similar regarding recipient age, recipient and donor gender, last donor creatinine, warm ischemia time, incidence of acute rejection during the first year post-kidney transplant.

**Table 1:** Demographic and relevant clinical information for the enrolled study patients separated according to deceased donor kidney undergoing PPP *vs.* CSP.

		PPP	CSP	
		Avg ± std^1^	Avg ± std	*p*-value
**N = **		60	39	
**Recipient Age**		49.6±13.0	50.9±13.8	0.63
**Recipient Gender (n)**	M (F)	37 (23)	23 (16)	0.16
**Recipient Race (%)**	AA (%)	61.7	58.9	0.130
**Recipient HCV status (pos, n)**		7	5	0.55
**CMV Disease (pos, n)**		0	0	N/A
**Donor Gender (n)**	M (F)	34 (26)	21 (18)	0.16
**Donor Race (%)**	AA (%)	30	41	0.18
**Donor Age (yrs.)**		42.0±14.6	34.1±14.2	**0.009**
**CIT (min)**		1367±509	1022±485	**<0.001**
**WIT/RVT (min)**		26.0±6.6	30.0±7.7	0.53
**DGF (%)**	% (n)	28.3 (17)	28.2 (11)	
**Acute Rejection Episodes (n)**		8	5	0.60
**PRA at transplant – T (%)**		43.1±37.7	37.7±35.8	0.48
**PRA at transplant – B (%)**		20.7±31.2	27.1±34.3	0.34
**Last eGFR (mL/min)** *		50.3±28.7	48.6±21.5	0.76

* eGFR at the end of the study for each patient (minimal follow-up 24 months post-transplantation).

Abbreviations: CIT = cold ischemia time, WIT/RVT = warm ischemia time/revascularization time, eGFR = estimated glomerular filtration rate, AA = African American, PRA = panel reactive antibodies, HCV = hepatitis C virus, CMV = cytomegalovirus, Estimated GFR (eGFR) was calculated using the abbreviated MDRD formula. P-values were calculated using Fisher's exact test.

1 All values are given as averages ± standard deviation if not otherwise stated.

Donor age was significantly different between groups (*P* = 0.009). The number of extended criteria donor (ECD) kidneys [5]–[7] as expected was higher in the PPP group (35% *vs.* 12.8%, *P* = 0.012). The donor age difference between groups relates with the higher number of ECD donor kidney in the PPP group.

Of interest, graft survival at the end of the study was similar between groups (88.5% in PPP group and 94.3% in CSP group (*P* = 0.2)). Moreover, the incidence of DGF was not statistically significant between PPP *vs.* CSP groups (28.3% (17 out of 60) *vs.* 28.2% (11 out of 39) (*P* = 0.18), respectively.

### Analysis of gene expression profiles between kidney preservation groups

**Figure 1:**
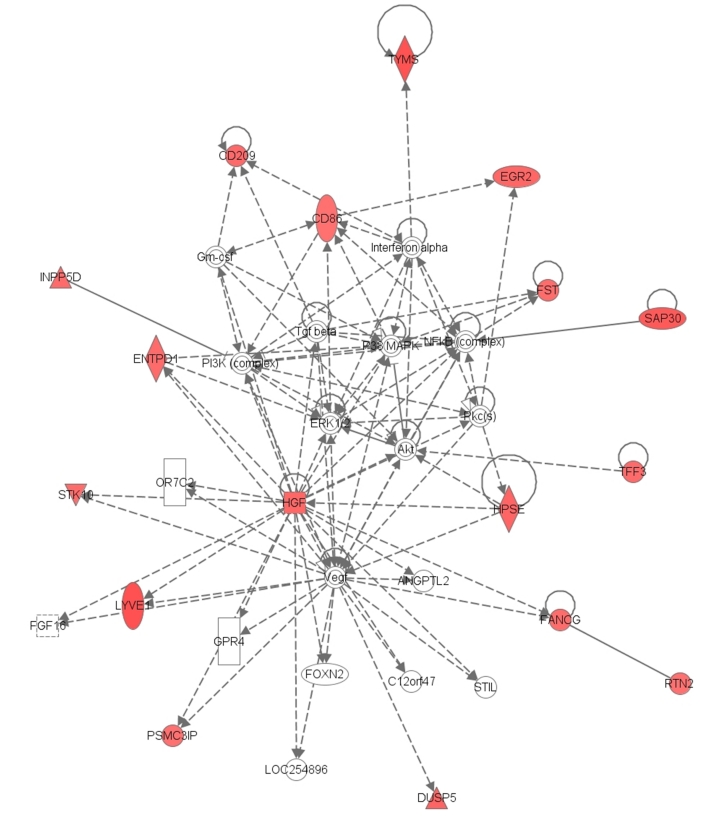
Top scoring network of differentially expressed genes between CSP and PPP kidneys. The top-scoring network of interactions among the probe sets identified as significantly differentially expressed when comparing CSP *vs.* PPP kidneys. The probe sets were subsequently analyzed using the Ingenuity Pathway Analysis software (https://analysis.ingenuity.com). This software is designed to identify dynamically generated biological networks, global canonical pathways and global functions. Interconnection of significant functional networks, where molecule nodes in different shades of red and green or white depending on being up-regulated and down-regulated or no-change, respectively, in CSP samples.

Forty-three probe sets were significantly different when comparing the PPP (n = 59) *vs.* CSP (n = 39) groups using a two-sample t-test and P<0.001. Core analysis was performed to interpret the data set in the context of biological processes, pathways and molecular networks. The associated functions to the top scored network (score 40) that related with the differentially expressed genes included antigen presentation and cell-to-cell signaling and interaction (**Figure 1**).

From the analysis of the genes differentially expressed between groups, genes involved in immune response (CD86, CD209, CLEC4, EGFR2, TFF3, INPP5D, among others) were significantly over-expressed in CSP kidneys. Moreover, the specific analysis of the over-expressed genes showed association with inflammation (ADORA3, CD86, CLEC4E, ENTPD1, HGF, IGHG1, IL11RA), cell movement of dendritic cells (CD209), activation of naïve T lymphocytes (CD86, IGHG1), pro-inflammatory response of macrophages (INPP5D), and binding and accumulation of macrophage (ENTPD1, INPP5D).

In the analysis of molecular and cellular functions' cell-to-cell signaling and interaction (*P* = 9.4E-05 to 4.8E-02) and antigen presentation (*P* = 1.2E-04 to 4.0E-02) were the more important functions of genes significantly over-expressed in CSP kidney samples. Seventeen probe sets were significantly different when adjusting the analysis for CIT using probe set level linear models (**Table 2**).

**Table 2:** Seventeen probe sets were significant when adjusting the analysis for cold ischemia time using probe set level linear models.

Affy ID	Gene Symbol	*P*-value
202589_at	TYMS	0.00074
203332_s_at	INPP5D	0.00083
203564_at	FANCG	0.00016
204493_at	BID	0.00078
204773_at	IL11RA	0.00008
205486_at	TESK2	0.00052
206230_at	LHX1	0.00092
207572_at	NA	0.00058
209457_at	DUSP5	0.00017
210064_s_at	UPK1B	0.00089
210755_at	HGF	0.00004
213566_at	RNASE6	0.00091
214770_at	MSR1	0.00030
219299_at	TRMT12	0.00100
219799_s_at	DHRS9	0.00078
34408_at	RTN2	0.00033
40420_at	STK10	0.00015

When the dataset was restricted to patients with a CIT<1200 minutes, there were 24 patients in PPP and 22 patients in CSP. There were 5 significant probe sets when comparing PPP *vs.* CSP groups using a two-sample t-test and *P*<0.001. There was still a significant difference with respect to CIT between these two groups (*P* = 0.004). Six probe sets were significant when adjusting the analysis for CIT using probe set level linear models

**Figure 2:**
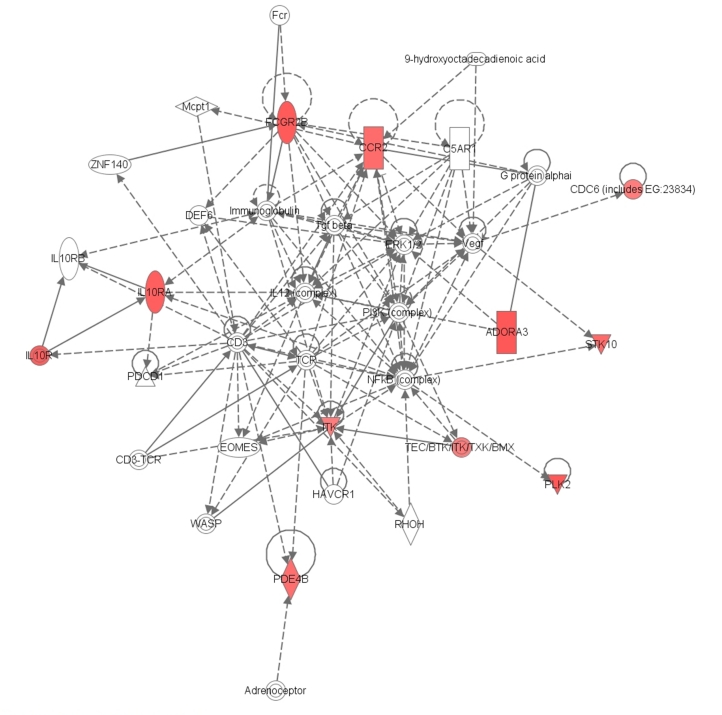
Differentially expressed genes between CSP and PPP kidneys with prolonged CIT. Eighteen probe sets were differentially expressed when CIT was higher than 1,200 minutes and not significant between groups (CSP *vs.* PPP). Pathway Analysis showed that almost all these genes belong to the same network (score 23) and associate with the inflammatory response and immune cell trafficking.

Importantly, when the dataset was restricted to patients with CIT>1200 minutes, there were 15 CSP and 37 PPP kidneys. Eighteen probe sets were significantly different when comparing the two groups (*P*<0.001) (**Table 3**) despite the fact that there was no significant CIT between groups (*P* = 0.08). Importantly, all the over expressed genes identified from this analysis, belong to the same network (score 23) (**Figure 2**). The biological functions associated with these genes were inflammatory response, cellular movement, and immune cell trafficking.

**Table 3:** Eighteen probe significantly different when the dataset was restricted to patients having a CIT>1200 minutes.

Affy ID	Gene Symbol	*P*-value
201939_at	PLK2	0.0005
203767_s_at	STS	0.0007
203968_s_at	CDC6	0.0007
204912_at	IL10RA	0.0004
206171_at	ADORA3	0.0001
206978_at	CCR2	0.0002
210064_s_at	UPK1B	0.0009
210644_s_at	LAIR1	0.0009
210889_s_at	FCGR2B	0.0003
211302_s_at	PDE4B	0.0004
211339_s_at	ITK	0.0006
213566_at	RNASE6	0.0006
215779_s_at	HIST1H2BG	0.0009
216348_at	RPS17P5	0.0007
217336_at	NA	0.0003
219414_at	CLSTN2	0.0008
219734_at	SIDT1	0.0010
40420_at	STK10	0.0002

### Gene expression profiles of expanded criteria donor kidneys (PPP vs. CSP)

ECD kidneys represented an important sub-group in our study population (27.7%) and in line with the reported use of these grafts in the USA.

**Figure 3:**
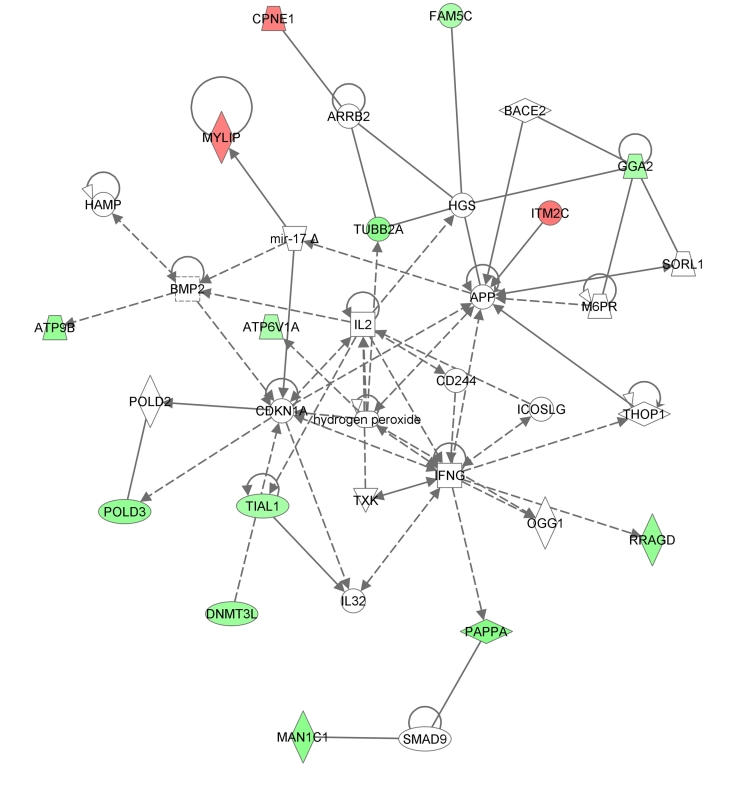
Inflammatory response in ECD-CSP kidneys. One-hundred and two differentially expressed probe sets were identified from the analysis of ECD kidneys preserved using PPP *vs.* CSP. From the gene list, the scoring network functions included inflammatory response and cell death (score 25) when using Pathway Analysis software.

Characteristics of the ECD kidneys by preservation group are shown in the **Table 4**. As expected, there were more ECD preserved with PPP. When gene expression profiles of ECD grafts (PPP *vs.* CSP) were compared, 102 probe sets were statistically differentially expressed (*P*<0.01). From the analysis of these genes, we observed that genes involved in inflammation were down-expressed in the ECD kidneys preserved with PPP as it is shown in the **Figure 3** (second top scored network, score 25). The incidence of DGF in ECD kidneys was higher but not statistically different in the PPP group (47.8% *vs.* 20%, *P* = 0.27 (one tailed Fisher Exact Probability Test). Moreover, there was not statistical significantly difference in graft function between ECD kidneys undergoing PPP or CSP at 24 months post-kidney transplantation.

**Table 4:** Characteristics of ECD* donor kidneys preserved using PPP and CSP.

	ECD-PPP n = 23	ECD-CSP N = 5	*P*-value
Recipient age (years)	50.2±12.6	56.8±4.5	0.27
Recipient race (%AA)	73.9	80.0	0.43
Recipient gender (%M)	65.2	60.0	0.29
Donor age (years)	46.5±16.3	49.8±14.6	0.69
Donor race (%AA)	26.0	40.0	0.48
Donor gender (%M)	73.9	80.0	0.39
CIT (minutes)	1,416±570	1,341±395	0.80
Last donor serum creatinine (mg/dL)	1.22±0.51	0.87±0.5	0.26
DGF (%)	(11/23) 47.8	(1/5) 20.0	0.22
eGFR at 24 months post-transplantation (mL/min)	57.2±30.3	51.2±7.6	0.67

* ECD defined as previously described [7].

AA: African-Americans, M: Male, DGF: Delayed Graft Function.

### Evaluation of gene expression in paired kidneys

**Figure 4:**
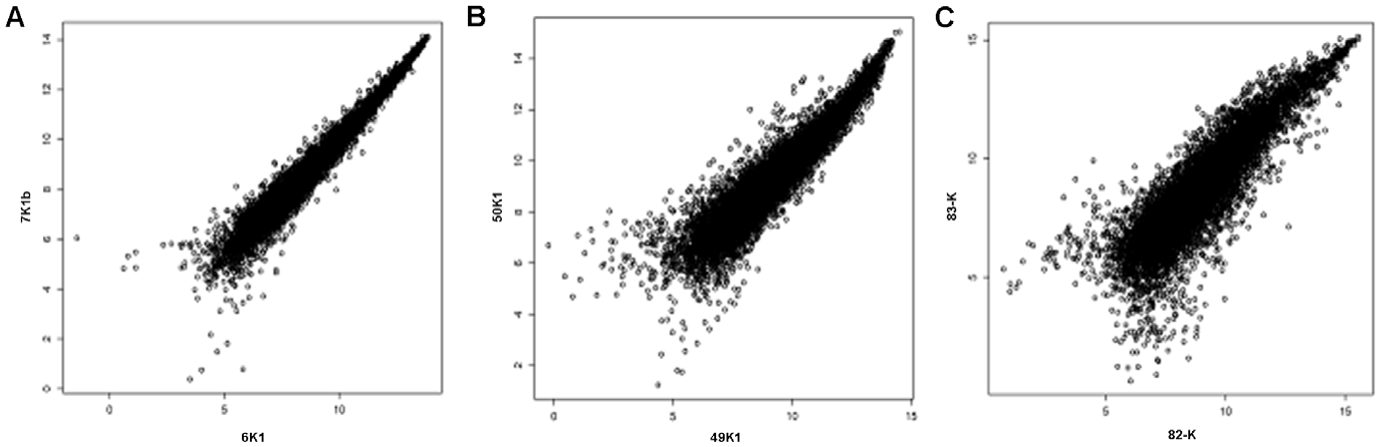
Paired kidney analyses. The S-Score method was applied to assess whether there were any differences in gene expression between paired kidneys. S-Score is a method that uses the probe level measurements in performing a test of hypothesis of differential probe set expression when only two GeneChips are available for each comparison. **A**) Set of paired SCD-PPP that did not developed DGF, **B**) Set of paired ECD-PPP with one kidney developing DGF post-transplantation (CIT not significant), **C**) Set of paired ECD-SCP kidney with one kidney developing DGF post-transplantation (CIT not significant).

In the present study, seven donors provided paired kidneys (14 kidneys). We also evaluated how the gene expression patterns differ among paired kidneys. **Figure 4** shows the gene expression profiles when two set of kidneys were compared at pre-implantation time using the S-Score method to test for differential expression between two different paired kidney sets. We specifically evaluated three paired kidneys including one set of SCD kidneys preserved using PPP and two sets of ECD kidneys preserved using PPP or CSP. The first set of SCD kidneys preserved using PPP performed well post-transplantation and the gene expression profiles were similar between samples at pre-implantation (**Figure 4A**). The second set of ECD undergoing PPP had one kidney developing DGF post-transplantation (even when CIT was not different between them) (**Figure 4B**). A similar situation was observed with the set of ECD kidneys preserved with CSP with only one kidney developing DGF post-transplantation (**Figure 4C**). Also, not differences in CIT were observed. As shown in **Figure 4**, a higher difference in probe sets was observed in the last set (ECD kidneys undergoing CSP, with one kidney developing DGF). For this last set of kidneys when applying the S-Score method to test for differential expression between samples and using the q-value method with q<0.05 to identify differentially expressed probe sets, 137 were identified. From the analysis of these genes, canonical pathway analysis showed that stress and tissue injury were down regulated in the paired kidneys that did not developed DGF post-kidney transplantation.

## Discussion

From the time a patient is identified as a potential organ donor it is critical to maintain adequate organ perfusion and avoid hypoxemia. In the 1970s, most of the donated kidneys were preserved by pulsatile perfusion preservation [23]. The situation had reversed in the 1980s with the majority of kidneys being preserved by cold storage preservation. The principal cause for the change in the organ preservation method was that large-scale studies of transplantation outcome [16]–[17], [24] failed to find any survival advantage for kidneys preserved by PPP. Consequently, the disadvantages of PPP, including the need for a usually large machine, consumables, technician and the risk of equipment failure, as compared with the simplicity and low cost of CSP, were not justified.

However, PPP has resurged in the last decade based upon the belief that this preservation method leads to a reduced rate of graft ischemia, DGF and eventually, improved long term graft function. At the same time, comparative studies between PPP and CSP started to show benefit of using PPP [24].

A report by Moers *et al*. [25] tends to support this practice. The authors reported a prospective multicenter analysis of 336 consecutive grafts from deceased donors. Paired kidneys were randomly divided and preserved by PPP or CSP and a total of 672 graft recipients were followed for 1 year. The authors reported lower DGF incidence in PPP grafts (*P* = 0.01) as well as a reduced serum creatinine level at 2 weeks post-kidney transplant and reduced risk of graft failure. Also, graft survival at 1 year was better in kidneys preserved by PPP (*P* = 0.04). As reported by the editorial in the same journal [26], the limitations of this study are that most of the kidney grafts had a short CIT (CIT mean 15 hr) and the lack of an advantage of the use of PPP in the subgroup analysis of ECD and donation after cardiac death kidneys.

Up-to- date, the published reports about the advantages and/or disadvantages of the use PPP over CSP [22]–[26] are observational and do not further explore the molecular and patho-physiological mechanisms leading to decreased incidence of DGF and better graft survival.

It is now well described that molecular profiling detects changes not seen by histological evaluation and clinical markers. However, the identification of predictive gene sets and the application to an individualized patient management needs the integration of clinical and pathology-based variables, as well as objective reference markers of graft function, post-transplant complications, and long-term outcomes [27].

At present, PPP is widely used in most areas of USA in renal transplantation, allowing a greater use of ECD kidneys and increasing the number of organs available for transplantation. In addition, perfusion parameters, such as flow rate and resistance are factors frequently used to assess and estimate the downstream functionality of renal grafts [28]. However, a better understanding of the molecular mechanisms involved in this complex process associated with ischemia reperfusion injury and graft function may provide biological insights supporting the recently described [25] benefit of the use of PPP.

To the best of our knowledge, this is the first report showing gene expression profiles of kidney donor biopsies in association with clinical/demographic donor and recipient characteristics to compare CSP *vs.* PPP in a prospective study.

Gene expression profile data of 99 consecutive deceased donor kidneys preserved with different methods (CSP *vs.* PPP) were evaluated. As expected, kidneys preserved by PPP had a tendency to have longer CIT; the analysis was therefore controlled for CIT. Our study included consecutive cadaveric donor kidney transplants that were performed following clinical/surgical team decisions based on organ quality despite the preservation method. The present study represents a realistic situation about the decisions and clinical practice that most regions in the USA challenge daily.

In the present cohort of patients, histological evaluation at pre-implantation time was performed for all the donor kidneys (**Table S1**). There were not statistical significant differences among groups. We did not identify relationships between glomerulosclerosis, tubular atrophy, and/or interstitial fibrosis and graft function with gene expression profiles. Moreover, these results are in concordance with the publication from Edwards *et al.* [29]. The authors showed, using cadaveric kidneys (n = 3,444) with reported biopsy results between October 25, 1999 and December 31, 2001, that glomerulosclerosis on donor kidney biopsies does not correlate well with 1-year graft survival and function, and percentage glomerulosclerosis should not be used as the sole criterion for discarding recovered cadaveric kidneys.

The principal finding from the molecular analysis was the presence of over-expression of inflammatory genes in CSP kidneys when compared with PPP kidneys. This is a point of critical interest as the PPP group demonstrated similar DGF incidence rates when compared with the CSP group despite of having significant higher number of ECD donors with an associated significant difference in donor age (older donors). In the last decade, the proportion of deceased donors older than 50 years of age has increased from 21% to 30% in USA [30], [31] making this issue critical for further evaluation in the kidney transplant field. With the knowledge that age alone is a determinant of DGF it is expected that ECD organs are at higher risk for complications than SCD organs [32]. A decreased level of inflammation associated with the use of PPP may explain these findings.

An imbalance in metabolic supply and demand within the ischemic organ results in profound tissue hypoxia and microvascular dysfunction. Subsequent reperfusion further enhances the activation of innate and adaptive immune responses and cell death programs. Recent advances in understanding the molecular and immunological consequences of ischemia and reperfusion may lead to innovative therapeutic strategies for treating patients with ischemia and reperfusion-associated tissue inflammation and organ dysfunction [33].

Our analysis demonstrated that the expression of CD209 gene was up-regulated in CSP kidneys. This gene encodes a transmembrane receptor and is often referred to as DC-SIGN because of its expression on the surface of dendritic cells and macrophages. Dendritic cells and macrophages play an important role in the innate and adaptive immune response of acute ischemia-reperfusion injury (IRI). In the kidney they reside in the interstitial extracellular compartment and are poised to interact with substances transported from the tubule lumen into peritubular capillaries, endogenous molecules released from parenchymal cells, exogenous invading organisms, or with resident or infiltrating immune cells including lymphocytes, nature killer T (NKT) cells, epithelial cells and fibroblasts. Dendritic cells and macrophages are key initiators, potentiators and effectors of innate immunity in kidney IRI and induce injury either through inflammatory signals to other effector cells or directly through the release of soluble mediators [34]. Activation of the innate immune system by ischemically damaged tissue may increase the production of chemokines and adhesion molecules by the endothelium and tubular epithelial cells to facilitate the entry of leukocytes into the kidney.

The expression of CD86 was also up-regulated in CSP kidneys. This protein is expressed by antigen-presenting cells, and it is the ligand for two proteins at the cell surface of T cells, CD28 antigen and cytotoxic T-lymphocyte-associated protein 4. Binding of this protein with CD28 antigen is a costimulatory signal for activation of the T-cell. Targeting the activation or effector function of lymphocytes is a potentially effective approach to treat or modify the immune response [34].

When the groups were sub-stratified according with CIT, kidneys with longer CIT (when no significance was observed in CIT between groups) presented an inflammatory signature when preserved with CSP. This finding demonstrates the effect of PPP in decreasing inflammation even on those kidneys with longer CIT.

From the evaluation of paired kidneys undergoing the same preservation method, we observed that two sets of paired ECD kidneys (one undergoing PPP (**Figure 4B**) and a second one CSP (**Figure 4C**) one kidney in each set developed DGF. However, when S-Score method was applied to evaluate the molecular differences between samples, a higher number of genes were differentially expressed between the ECD kidneys undergoing CSP with down regulation of stress and tissue injury in the kidney that did not develop DGF.

Biological modulation of ischemic acute kidney injury aims to reduce the incidence of delayed graft function and to safely increase the number of kidney transplantations using organs that have suffered prolonged warm and cold ischemia [35]. In the present article, we observed that the more important differential pattern associated with pre-implantation biopsies when CSP and PPP were compared, was inflammation. This pathway was up-regulated in CSP kidneys even when the PPP group has a large number of ECD and donor after cardiac death kidneys. Moreover, no significant difference was observed in DGF incidence and graft function post-transplantation (minimal follow-up two years). The importance of this study relies not only in the identification of the molecular signatures that characterize the use of PPP *vs.* CSP and the sub-analysis per donor type (ECD *vs.* SCD) but also in the functional validation of the results. All the studied biopsies were from kidney grafts that were transplanted in recipients with end stage kidney disease and followed for at least 24 months post-transplantation. The present findings, based in the study of an important number of samples and patient characterization, support the importance of the use of PPP in ECD donor kidneys.

## Supporting Information

Table S1 Histological evaluation of pre-implantation biopsies classified by sub-groups.
(DOC)
