# Supplementary material for: Reduced Expression of Inflammatory Genes in Deceased Donor Kidneys Undergoing Pulsatile Pump Preservation
Source: PLoS One. 2012 Apr 24;7(4):e35526. doi: 10.1371/journal.pone.0035526 (PMC3335841; doi:10.1371/journal.pone.0035526)
Supplement: Table S1 — Histological evaluation of pre-implantation biopsies classified by sub-groups. (DOC) [file pone.0035526.s001.doc]

**Table S1-** Histological evaluation of pre-implantation biopsies classified by sub-groups

| **Patient Group** | **score** | **GSC** | **IF** | **TA** |
| --- | --- | --- | --- | --- |
| PPP (number of patients) | 0 | 42 | 25 | 32 |
|  | 1 | 12 | 28 | 21 |
|  | 2 | 1 | 2 | 2 |
|  | 3 | 0 | 0 | 0 |
|  | N/A | 5 | 5 | 5 |
|  | total | 60 | 60 | 60 |
| CSP (number of patients) | 0 | 25 | 24 | 29 |
|  | 1 | 10 | 10 | 5 |
|  | 2 | 1 | 2 | 2 |
|  | 3 | 0 | 0 | 0 |
|  | N/A | 3 | 3 | 3 |
|  | total | 39 | 39 | 39 |

GS: glomerulosclerosis, IF: Interstitial fibrosis, TA: Tubular atrophy, N/A: information non- available
